# Associations of two-pore domain potassium channels and triple negative breast cancer subtype in *The Cancer Genome Atlas*: systematic evaluation of gene expression and methylation

**DOI:** 10.1186/s13104-017-2777-4

**Published:** 2017-09-12

**Authors:** Keith A. Dookeran, Wei Zhang, Leslie Stayner, Maria Argos

**Affiliations:** 1 Epidemiology, University of Wisconsin-Milwaukee, Joseph J. Zilber School of Public Health, 1240 N. 10th St, Milwaukee, WI 53205 USA; 2 The Cancer Foundation for Minority and Underserved Populations, Chicago, IL 60614 USA; 3 Department of Preventive Medicine, Northwestern University Feinberg School of Medicine, 680 N. Lake Shore Drive, Suite 1400, Chicago, IL 60611 USA; 4 Division of Epidemiology and Biostatistics, University of Illinois at Chicago, School of Public Health, 1603 West Taylor Street, MC923, Chicago, IL 60612 USA

**Keywords:** Potassium channels, Breast cancer, Subtype, TCGA

## Abstract

**Objectives:**

It is unclear whether 2-pore domain potassium channels are novel molecular markers with differential expression related to biologically aggressive triple-negative type breast tumors. Our objective was to systematically evaluate associations of 2-pore domain potassium channel gene expression and DNA methylation with triple-negative subtype in *The Cancer Genome Atlas* invasive breast cancer dataset. Methylation and expression data for all fifteen 2-pore domain potassium family genes were examined for 1040 women, and associations with triple-negative subtype (vs. luminal A) were evaluated using age/race adjusted generalized-linear models, with Bonferroni-corrected significance thresholds. Subtype associated CpG loci were evaluated for functionality related to expression using Spearman’s correlation.

**Results:**

Overexpression of *KCNK5*, *KCNK9* and *KCNK12*, and underexpression of *KCNK6* and *KCNK15*, were significantly associated with triple-negative subtype (Bonferroni-corrected p < 0.0033). A total of 195 (114 hypomethylated and 81 hypermethylated) CpG loci were found to be significantly associated with triple-negative subtype (Bonferroni-corrected p < 8.22 × 10^−8^). Significantly negatively correlated expression patterns that were differentially observed in triple-negative vs. luminal A subtype were demonstrated for: *KCNK2* (gene body: cg04923840, cg13916421), *KCNK5* (gene body: cg05255811, cg18705155, cg09130674, cg21388745, cg00859574) and *KCNK9* (TSS1500: cg21415530, cg12175729; *KCNK9/TRAPPC9* intergenic region: cg17336929, cg25900813, cg03919980). CpG loci listed for *KCNK5* and *KCNK9* all showed relative hypomethylation for probability of triple-negative vs. luminal A subtype. Triple-negative subtype was associated with distinct 2-pore domain potassium channel expression patterns. Both *KCNK5* and *KCNK9* overexpression appeared to be functionally related to CpG loci hypomethylation.

**Electronic supplementary material:**

The online version of this article (doi:10.1186/s13104-017-2777-4) contains supplementary material, which is available to authorized users.

## Introduction

Two-pore domain potassium (K2p) channels enable background leak of potassium (K+) ions and the K2p-family has 15 members [1, 2]. K2p-channels are important for baseline cellular activity at rest including membrane-potential, calcium homeostasis and cell-volume regulation [3]. Evidence from laboratory studies supports the hypothesis that alterations in expression/function of K2p-channels may play a role in cancer development/progression [3–5]. The role of K2p-channels in breast cancer (BC) is emerging and recent reviews suggest potential clinical utility [4, 6, 7]. Williams et al. examined K2p-channel expression in a microarray database study, and reported that all but five members showed altered expression in BC [3].

Preliminary reports suggest that some K2p-channels may be novel molecular markers with differential expression related to biologically-aggressive triple-negative (TN) type BC. In estrogen-receptor-alpha positive (ER+) cell lines, *KCNK5* expression appears to be under regulatory control of ER-alpha, and estrogen-response-elements (ERE) are found in the enhancer region of *KCNK5* [8]. Clarke et al. suggest that upregulation of *KCNK5* is associated with poor outcome for TNBC-related basal-like subtype [9]. Conway et al. suggest that *KCNK4* may be differentially methylated according to race, with relatively higher median DNA-methylation observed for non-Hispanic (nH) black vs. nH-white BC [10]. Other studies similarly suggest that differential methylation of *KCNK9* may be related to nH-black race and TNBC [11–14]. Mu et al. first described the *KCNK9* gene on 8q24.3 as a potential proto-oncogene; gene-amplification (10%) and protein-overexpression (44%) were detected in BC, but not in normal tissue-controls [15]. DNA copy-number loss has been observed for *KCNK12* in a small clinical study of tubular BC [16].

K2p-channels also hold promise for innovative oncologic therapeutic/prevention strategies [7, 17, 18]. Current molecular-epidemiologic data characterizing K2p-channels in clinical BC are limited. Our study goal was to systematically evaluate associations between K2p-gene-family mRNA-expression and DNA-methylation with TNBC in *The*-*Cancer*-*Genome*-*Atlas* (TCGA), which contains genomic information from DNA-methylation and mRNA-expression arrays for almost 1100 human BC.

## Main text

### Materials and methods

#### Sample and procedure

Publicly available BC data was downloaded/aggregated (KAD) from *TCGA*-*Data*-*Matrix* and *cBioPortal*-*for*-*Cancer*-*Genomics* (provisional dataset) web-sites (February 2016) [19, 20]. TCGA molecular BC dataset was generated by *TCGA*-*Research*-*Network* and details have been previously published [21, 22]. The assembled dataset of invasive BC (using TCGA sample-code suffix −01 for primary solid-tumors) included information on: (1) clinical-pathological factors: age/stage at diagnosis, menopausal status, lymph-node status, estrogen/progesterone receptor (ER/PR) status, human-epidermal-growth-factor-receptor-2 (HER2) status, and race/ethnicity; and (2) K2p-gene-markers including: (a) Level 3 DNA-methylation probes (*Illumina*-*Infinium*-*Human*-*Methylation*-*450K*-*BeadChip* beta-values (450K array), n = 767) [23]; and (b) Level 3 RNA-Seq gene-expression data (*Illumina*-*HiSeq*-*platform*, gene level RNA-Seq by Expectation Maximization (RSEM)-normalized and log2-transformed values, n = 959) [24]. Cytosine–guanine (GpG) methylation loci within 25 kb from either end of the genes of interest were included for examination (*UCSC*-*Genome*-*Browser* [25], hg37); 724 GpG loci were included, but was reduced to 608 after exclusion of probes with null-reads. Analytic dataset consisted of 1040 women with information on stage, and all women with available data were included in analyses. The study was approved by UIC IRB.

#### Breast cancer subtype

The primary study-endpoint was a binary variable of combined TN-status, developed by aggregating data from immunohistochemical (IHC) and intrinsic subtype assays (PAM50) with a referent of luminal-A status (ER/PR+/HER2− status). For sensitivity analyses, data on *Intrinsic Subtype* classification was obtained from supplementary publication data based on PAM50 assay [21], and data on *Integrative Cluster* (IC) classification based on copy-number-change was obtained from *Cancer*-*Research*-*UK*-*Cambridge*-*Institute* (Oscar Rueda and Carlos Caldas) [26–28]. Secondary endpoints in sensitivity analyses included: ER/PR− status (IHC; referent ER/PR+); basal-subtype (PAM50; ER/PR−/HER2−, and cytokeratin 5/6+ and/or HER1+; referent luminal-A); and IC10-type (referent IC3). Gene-expression patterns were also compared for RSEM and Agilent arrays.

#### Statistical methods

For categorical variables, Chi square test-of-association was used to examine significance of relationships. For continuous variables, t-test was used to compare means as appropriate to distribution characteristics; and for non-normally distributed data, nonparametric Wilcoxon rank-sum test was used.

Tertiles were used for categorization of gene-expression, except where limited by small sample-signals, then binary mRNA-overexpression was defined as a z-score > +1 standard deviation (SD) [29]. Association of all K2p gene-expression and DNA-methylation with TN-subtype was evaluated using age (years) and race (nH-white, nH-black) adjusted generalized-linear regression-models (glm) with binomial-distribution and log-link to estimate prevalence risk-ratios (RRs) and their corresponding 95% confidence-intervals (CIs). The significance threshold for gene-expression and DNA-methylation was p < 0.05 and Bonferroni-corrected p-values were used where appropriate (Bonferroni-adjusted threshold for expression was p < 0.0033; and for methylation p < 8.22 × 10^−8^).

Methylation glm results were sorted on p-values (smallest to largest) and all loci associated with TN-subtype were selected for reporting and further analysis; loci were then evaluated for functionality related to expression using Spearman’s correlation. Methylation was deemed significantly correlated with expression if the Spearman’s Rho-value was either ≤−0.2 or ≥+0.2 and associated with a p-value <0.05 [10].

## Results

Characteristics of the study sample are presented in Table 1. Overall, most of the sample was age >50 years (73%), postmenopausal (76%), nH-white (86%), stage-II (58%), and ER/PR+ (66%) and HER2− (89%); 631 patients had data on TN (n = 159) vs. luminal-A (n = 472) status.

**Table 1:** Selected characteristics of study sample

Characteristic	Gene expression^a^		Methylation^b^	
	N	[%]	N	[%]
Total	959	[100]	767	[100]
Race				
nH white	592	[85.6]	471	[78.6]
nH black	100	[14.5]	128	[21.4]
Age (years)				
>/=50	682	[73.3]	477	[71.3]
<50	248	[26.7]	192	[28.7]
Menopausal status				
Post	653	[76.1]	497	[75]
Pre	205	[23.9]	165	[25]
Stage				
I	160	[17.3]	126	[17.4]
II	536	[58]	402	[55.4]
III	213	[23.1]	188	[25.9]
IV	15	[1.6]	10	[1.4]
HR expression				
ER+/PR+	585	[65.6]	452	[65.8]
ER+/PR−	104	[11.7]	78	[11.4]
ER−/PR+	14	[3.3]	15	[2.2]
ER−/PR−	189	[21.2]	142	[20.7]
Intrinsic subtype				
Luminal A	219	[45]	108	[51.4]
Luminal B	116	[23.9]	43	[20.5]
HER2+/HR−	55	[11.3]	14	[6.7]
Basal-like	88	[18.1]	40	[19]
Normal-like	8	[1.7]	5	[2.4]
Combined TN status endpoint				
Luminal A	472	[74.8]	398	[76.7]
Triple negative	159	[25.2]	121	[23.3]

*nH* non-Hispanic, *HR* hormone receptor, *ER/PR* estrogen and progesterone receptor, *HER2* human epidermal growth factor receptor-2

To demonstrate the basic distribution of study covariates:

^a^An expression ‘flag’ marker (KCNK2) with no missing values is used (mRNA Expression z-scores, RNA Seq V2 RSEM)

^b^A methylation ‘flag’ marker (cg21415530, KCNK9) with no missing values is used

All K2p gene-expression was non-normally distributed (Additional file 1: Table S1). Table 2 demonstrates that overexpression of *KCNK5*/*KCNK9*/*KCNK12*, and underexpression of *KCNK6*/*KCNK15*, were associated with TN-subtype in age/race adjusted models (all p < 0.0033). In fully-adjusted models, *KCNK5*/*KCNK9*/*KCNK12*/*KCNK15* remained associated with TN-subtype.

**Table 2:** Association of K2p gene expression and triple negative subtype

	Age/race adjusted^a^				Mutually adjusted^b^				Fully adjusted^c^			
	RR	95% CI		p-value^d^	RR	95% CI		p-value^e^	RR	95% CI		p-value^e^
KCNK1	1.02	0.94	1.10	6.50E−01	…	…	…	…	…	…	…	…
KCNK2	0.88	0.63	1.22	4.28E−01	…	…	…	…	…	…	…	…
KCNK3	0.95	0.74	1.24	7.25E−01	…	…	…	…	…	…	…	…
KCNK4	1.02	0.94	1.10	6.66E−01	…	…	…	…	…	…	…	…
KCNK5	1.03	1.02	1.04	*2.92E−09*	2.51	1.88	3.36	*4.26E−10*	2.22	1.67	2.96	*3.81E−08*
KCNK6	0.24	0.17	0.33	*1.38E−17*	0.87	0.76	0.99	2.96E−02	0.88	0.77	1.01	7.68E−02
KCNK7	0.97	0.90	1.05	5.09E−01	…	…	…	…	…	…	…	…
KCNK9	1.02	1.01	1.03	*1.43E−03*	1.18	1.08	1.30	*2.91E−04*	1.16	1.06	1.28	*2.39E−03*
KCNK10	1.06	1.00	1.13	4.44E−02	…	…	…	…	…	…	…	…
KCNK12	1.03	1.02	1.04	*8.93E−09*	1.54	1.36	1.74	*4.60E−12*	1.54	1.35	1.76	*1.74E−10*
KCNK13	0.90	0.74	1.08	2.57E−01	…	…	…	…	…	…	…	…
KCNK15	0.0036	0.00	0.01	*2.21E−19*	0.28	0.20	0.38	*4.44E−16*	0.27	0.19	0.38	*2.86E−14*
KCNK16	0.0199	0.00	5.31	1.69E−01	…	…	…	…	…	…	…	…
KCNK17	1.03	0.91	1.16	6.32E−01	…	…	…	…	…	…	…	…
KCNK18	1.03	0.96	1.10	4.55E−01	…	…	…	…	…	…	…	…

mRNA Expression z-Scores (RNA Seq V2 RSEM)

Reference group for triple negative subtype is luminal A

*K2p* 2-Pore domain potassium channel, *ellipses* not applicable

^a^Models adjusted for race and age, and K2p gene expression is modeled as individual continuous covariates (n = 482)

^b^Models adjusted for race, age and other selected K2p gene expression variables as continuous covariates, while main K2p gene of interest is modeled as categorical tertiles (n = 482); only significant K2p gene expression from univariate glm included

^c^Fully adjusted model adds stage as an ordinal variable and menopausal status as a binary variable to the mutually adjusted mode; only significant K2p gene expression from univariate glm included

^d^From logistic regression models (glm), Bonferroni adjusted significance threshold = p < 0.0033 (significant values are in italics)

^e^From glm models p-trend, Bonferroni adjusted significance threshold = p < 0.0033

Gene-expression sensitivity analyses (Additional file 2: Table S2) show similar K2p RSEM-expression patterns for ER/PR-, basal and IC10 type tumors; however, overexpression of *KCNK7* was also seen with basal-subtype, and expression of *KCNK9* and *KCNK5* was not associated with basal and IC10 types, respectively. Comparative analyses using the Agilent expression-array (n = 590) (Additional file 2: Table S2) shows similar expression patterns for *KCNK5*/*KCNK6*/*KCNK12*/*KCNK15*, but additionally shows consistent underexpression of *KCNK4* across subtype, and that *KCNK9* was not associated with any subtype for this array.

Of 608 CpG loci, 195 (114 hypomethylated and 81 hypermethylated) loci were found to be associated with TN-subtype (p < 8.22 × 10^−8^, Additional file 3: Table S3). Significantly-associated negatively-correlated expression patterns differentially observed in TN vs. luminal-A subtype (Table 3; and Additional file 4: Table S4) were demonstrated for: *KCNK2* (gene-body: cg04923840, cg13916421), *KCNK5* (gene-body: cg05255811, cg18705155, cg09130674, cg21388745, cg00859574) and *KCNK9* (TSS1500: cg21415530, cg12175729; *KCNK9/TRAPPC9* intergenic: cg17336929, cg25900813, cg03919980). Loci listed for *KCNK5*/*KCNK9* all showed relative hypomethylation for probability of TN vs. luminal-A subtype.

**Table 3:** Annotation and expression correlation for select K2p methylation loci associated with triple negative subtype

Gene	GLM Models^a^		Annotation^b^	Luminal A^c^			Combined triple negative^c^		
CpG loci	Beta	p-value		N	Rho	p-value	N	Rho	p-value
KCNK1									
cg17851113	0.188	1.45E−12		339	*0.487*	*1.31E−21*	103	*0.301*	*2.02E−03*
cg23054119	−0.140	3.40E−06		338	*0.498*	*1.37E−22*	103	−*0.114*	*2.53E−01*
KCNK2									
cg04923840	−0.219	2.00E−25	Body	339	*0.227*	*2.52E−05*	103	−*0.253*	*9.81E−03*
cg06873024	−0.183	1.64E−21	Between CENPF/KCNK2	339	0.124	2.21E−02	103	*0.241*	*1.43E−02*
cg24464500	−0.206	9.81E−20	Body	337	*0.280*	*1.70E−07*	103	−0.048	6.33E−01
cg13916421	0.085	1.53E−11	Body	339	0.061	2.60E−01	103	−*0.211*	*3.22E−02*
cg00848374	0.129	2.35E−07	1stExon; 5′UTR	338	*0.202*	*1.83E−04*	103	−0.079	4.27E−01
KCNK3									
cg20491914	−0.170	2.97E−11	TSS1500	339	−*0.297*	*2.46E−08*	103	−0.164	9.82E−02
cg11273176	−0.128	1.60E−10	Body	339	−*0.388*	*1.23E−13*	103	−*0.408*	*1.88E−05*
cg06854842	−0.090	2.20E−09	Body	339	−*0.360*	*7.91E−12*	103	−*0.451*	*1.78E−06*
cg05616379	0.078	1.50E−06	Body	339	−*0.221*	*3.97E−05*	103	−*0.452*	*1.64E−06*
KCNK4									
cg01708924	−0.185	2.87E−31	3′UTR PRDX5	339	−*0.221*	*3.92E−05*	103	0.017	8.68E−01
cg10718809	−0.148	2.81E−23	PRDX5: body	339	−*0.224*	*3.04E−05*	103	−0.026	7.98E−01
cg10155572	−0.037	1.92E−07	ESRRA: body	339	−*0.239*	*8.75E−06*	103	−0.001	9.92E−01
KCNK5									
cg05255811	−0.373	9.32E−70	Body	339	−*0.369*	*2.40E−12*	103	−*0.674*	*5.95E−15*
cg18705155	−0.226	2.71E−26	Body	339	−0.120	2.78E−02	103	−*0.605*	*1.33E−11*
cg09130674	−0.224	1.52E−25	Body	339	−0.197	2.65E−04	103	−*0.711*	*3.91E−17*
cg21388745	−0.119	1.81E−23	Body	339	−*0.203*	*1.68E−04*	103	−*0.701*	*1.59E−16*
cg00859574	−0.129	4.14E−08	Body	339	−0.160	3.04E−03	103	−*0.430*	*5.90E−06*
cg02128567	−0.113	3.53E−07	1stExon; 5′UTR	339	−*0.253*	*2.35E−06*	103	−*0.345*	*3.62E−04*
KCNK6									
cg13521973	0.039	6.36E−07	Body	339	−*0.244*	*5.62E−06*	103	−0.193	5.07E−02
cg08216899	−0.025	5.10E−06	3′UTR	339	0.110	4.25E−02	103	*0.217*	*2.75E−02*
KCNK7									
cg10142520	−0.210	3.27E−22	EHBP1L1 body	339	−*0.215*	*6.77E−05*	103	0.014	8.90E−01
cg17290213	−0.126	1.09E−17	EHBP1L1 body	339	−*0.223*	*3.57E−05*	103	0.092	3.55E−01
cg03416228	−0.092	6.06E−15	Between EHBP1L1/KCNK7	339	−*0.225*	*2.91E−05*	103	−0.171	8.44E−02
cg12758867	−0.072	2.02E−14	EHBP1L1 body	339	−*0.223*	*3.49E−05*	103	0.095	3.38E−01
cg13179915	−0.067	3.13E−11	1stExon	339	−*0.205*	*1.39E−04*	103	−*0.305*	*1.75E−03*
cg05436845	−0.036	6.28E−06	MAP3K11: body	339	−*0.223*	*3.34E−05*	103	0.045	6.51E−01
cg17918700	−0.030	1.55E−05	TSS200	339	−*0.230*	*1.84E−05*	103	−*0.277*	*4.55E−03*
KCNK9									
cg21415530	−0.332	1.14E−56	TSS1500	339	−0.159	3.32E−03	103	−*0.383*	*6.39E−05*
cg12175729	−0.273	6.23E−22	TSS1500	339	−0.056	3.02E−01	103	−*0.215*	*2.88E−02*
cg20761810	0.155	1.67E−16	Between KCNK9/TRAPPC9	339	*0.238*	*9.24E−06*	103	−0.119	2.30E−01
cg05988964	0.183	2.15E−11	3′UTR	339	*0.208*	*1.16E−04*	103	0.197	4.56E−02
cg17336929	−0.110	9.79E−10	Between KCNK9/TRAPPC9	339	0.055	3.13E−01	103	−*0.248*	*1.17E−02*
cg25900813	−0.106	4.38E−08	Between KCNK9/TRAPPC9	339	0.069	2.07E−01	103	−*0.236*	*1.62E−02*
cg24020826	0.102	7.26E−08	3′UTR	339	0.176	1.12E−03	103	*0.204*	*3.84E−02*
cg18195416	0.125	6.90E−06	Between COL22A1/KCNK9	339	*0.206*	*1.29E−04*	103	*0.237*	*1.60E−02*
cg03919980	−0.066	2.96E−05	Between KCNK9/TRAPPC9	339	0.114	3.59E−02	103	−*0.222*	*2.43E−02*
KCNK10									
cg09945147	0.219	3.03E−33	Between GPR65/KCNK10	339	*0.241*	*7.35E−06*	103	*0.312*	*1.32E−03*
cg01733928	0.153	4.71E−29	Between GPR65/KCNK10	339	*0.250*	*3.17E−06*	103	*0.282*	*3.97E−03*
cg08069902	0.190	9.47E−26	Body	339	0.088	1.07E−01	103	*0.231*	*1.90E−02*
cg02222791	0.225	1.04E−23	Between GPR65/KCNK10	339	*0.238*	*9.06E−06*	103	*0.267*	*6.42E−03*
cg18078958	0.219	2.22E−20	Between GPR65/KCNK10	339	0.181	8.11E−04	103	*0.290*	*2.94E−03*
cg10172979	0.123	1.23E−14	Between GPR65/KCNK10	339	0.167	2.01E−03	103	*0.286*	*3.40E−03*
cg24740404	0.167	1.05E−13	Between GPR65/KCNK10	339	0.166	2.12E−03	103	*0.211*	*3.21E−02*
cg15347348	0.134	2.77E−13	Body; TSS1500	339	0.118	2.99E−02	103	*0.313*	*1.27E−03*
cg19453093	0.081	3.20E−12	Body	339	*0.205*	*1.46E−04*	103	*0.375*	*9.54E−05*
cg19476426	0.145	2.58E−11	Between GPR65/KCNK10	339	0.172	1.44E−03	103	*0.212*	*3.16E−02*
cg15493607	0.167	4.53E−11	Body; TSS1500	339	0.197	2.64E−04	103	*0.313*	*1.29E−03*
cg17671157	−0.105	6.87E−10	1stExon; 5′UTR	337	−0.068	2.10E−01	103	*0.229*	*1.97E−02*
cg18525616	0.144	8.98E−10	Between GPR65/KCNK10	339	0.156	4.04E−03	103	*0.270*	*5.81E−03*
cg00927624	0.143	3.91E−09	Between KCNK10/SPATA7	339	*0.249*	*3.43E−06*	103	*0.318*	*1.08E−03*
cg23291854	−0.107	4.26E−08	1stExon; 5′UTR	339	−0.040	4.65E−01	103	*0.282*	*3.85E−03*
cg22521269	−0.093	1.35E−06	TSS200	339	−0.116	3.35E−02	103	*0.215*	*2.89E−02*
cg02883668	0.079	2.03E−05	Body	339	0.177	1.07E−03	103	*0.262*	*7.40E−03*
cg23381267	0.097	6.14E−05	Body	339	0.154	4.44E−03	103	*0.227*	*2.11E−02*
KCNK12									
cg00981060	0.183	1.55E−20	Between MSH2/KCNK12	339	0.073	1.79E−01	103	*0.223*	*2.38E−02*
cg27138584	−0.059	4.47E−05	Body	339	0.003	9.63E−01	103	*0.258*	*8.46E−03*
cg00783525	0.054	7.26E−05	Body	339	0.113	3.70E−02	103	*0.335*	*5.39E−04*
KCNK13									
cg21191365	−0.209	2.20E−20	Body	339	−0.026	6.39E−01	103	*0.201*	*4.17E−02*
cg00364611	−0.118	1.32E−11	1stExon	339	−0.170	1.66E−03	103	*0.275*	*4.87E−03*
cg25225073	−0.100	4.05E−07	Body	339	0.073	1.80E−01	103	*0.303*	*1.88E−03*
KCNK15									
cg13598409	0.210	1.39E−56	TSS1500	339	−*0.251*	*2.87E−06*	103	0.093	3.50E−01
cg04966972	0.164	7.67E−45	TSS1500	339	−*0.298*	*2.14E−08*	103	0.078	4.35E−01
cg11681959	0.109	5.52E−28	TSS200	339	−*0.278*	*2.01E−07*	103	−0.053	5.98E−01
cg25301532	−0.113	6.12E−09	Body	339	*0.253*	*2.35E−06*	103	*0.290*	*2.99E−03*
cg09357268	−0.048	4.75E−08	Body	339	0.195	2.96E−04	103	*0.225*	*2.24E−02*
KCNK16									
cg05897803	0.114	3.86E−11	TSS200	339	−*0.342*	*1.04E−10*	103	0.009	9.28E−01
cg07970874	0.087	2.82E−06	TSS200	339	−*0.269*	*4.73E−07*	103	0.019	8.47E−01
KCNK17									
cg13855924	−0.142	2.45E−12	Body	339	−*0.209*	*1.09E−04*	103	−0.105	2.93E−01
cg04755571	−0.131	5.17E−10	TSS200	339	−*0.241*	*7.29E−06*	103	−*0.262*	*7.62E−03*
cg06347083	−0.124	5.92E−10	TSS200	339	−*0.215*	*6.61E−05*	103	−*0.216*	*2.82E−02*
cg10712551	−0.122	1.58E−07	Body	339	−0.175	1.19E−03	103	−*0.239*	*1.49E−02*
cg03252829	−0.105	1.61E−05	1stExon; 5′UTR	339	−*0.284*	*1.04E−07*	103	−*0.200*	*4.28E−02*
cg08315770	−0.103	6.55E−05	1stExon	339	−*0.205*	*1.48E−04*	103	−0.084	4.02E−01

mRNA expression z-scores (RNA Seq V2 RSEM)

Spearman’s Rho values of <0.2 or >0.2 were considered evidence of correlation if also associated with p-values <0.05 (these values are in italics)

*K2p* 2-pore domain K+ channel genes, *glm* generalized linear model

^a^Age and race adjusted associations of individual methylated CpG loci and combined triple negative vs. luminal A subtype were ranked according to p-value (smallest first) and all significant loci were selected for further evaluation (Bonferroni adjusted threshold for methylation analyses = p < 8.22 × 10(−8))

^b^Annotation data obtained from Illumina for Human Methylation 450K chip array (http://support.illumina.com/array/array_kits/infinium_humanmethylation450_beadchip_kit/downloads.html)

^c^Functional correlation between methylation and expression was then examined using Spearman’s correlation and results are presented stratified by subtype

## Discussion

K2p RSEM-expression analyses show that specific genes are associated with TN-subtype (overexpression of *KCNK5*/*KCNK9*/*KCNK12*, and underexpression of *KCNK6*/*KCNK15)*. Sensitivity analyses demonstrate consistency; similar RSEM-expression patterns of association were largely evident for ER/PR-, basal and IC10 related subtypes, although *KCNK9* and *KCNK5* overexpression failed to meet significance criteria for basal and IC10 types, respectively. Comparative analyses with the Agilent-array supports RSEM findings for *KCNK5/KCNK6/KCNK12*/*KCNK15*.

Regarding specificity for association of K2p-expression with tumor vs. normal sample-type, we used the *MEXPRESS web tool for visualizing expression, DNA methylation and clinical TCGA data* [30], and found that *KCNK9* (p = 2.58 × 10^−9^) and *KCNK12* (p = 4.63 × 10^−10^) overexpression appeared to be associated with tumor type, while *KCNK5* overexpression appeared to have marginal association (p = 0.0757). *KCNK6* and *KCNK15* overexpression (associated in our study with luminal-A subtype) also appeared to be associated with tumor sample-type (both p < 2.2 × 10^−16^).

Specific methylation loci on *KCNK2*/*KCNK5*/*KCNK9* were found to be significantly-associated with negatively-correlated gene-expression and differentially observed in TN vs. luminal-A subtype, which suggests potential functional significance. All select loci on *KCNK5*/*KCNK9* had negative delta-beta values indicating relative hypomethylation in TN-subtype. The overall levels of negative expression Rho-values for *KCNK5* and *KCNK9* were more than twice larger for TN than luminal-A subtype. Hence it is plausible that *KCNK5*/*KCNK9* overexpression related to TN-subtype may be functionally related to specific tumor CpG loci hypomethylation.

Prior literature suggests *KCNK5* may be under control of ER-alpha signaling and upregulation is associated with worse prognosis for basal-type tumors [8, 9]. Although we found association of *KCNK5*/*KCNK9* overexpression and TN-subtype, we were unable to relate to prognosis. Further, 17-beta-estradiol is known to induce *KCNK5* gene-expression via EREs found in the enhancer region of the *KCNK5* gene [8]. Hence the observed pattern in our study could be interpreted as evidence of loss of regulatory control of ER-alpha signaling pathways. An alternate explanation could be that *KCNK5* gene-expression in TNBC is incompletely understood. Prior studies have implicated *KCNK9* as a proto-oncogene in BC and suggest importance in TNBC [12–15, 31]. *KCNK9* is a maternally-imprinted gene with monoallelic-expression predominantly in brain tissue, but expression has been observed in breast tissue, and both are of ectodermal origin [11]. It is theorized that *KCNK9* overexpression may occur due to acquired relative hypomethylation and subsequent functionally biallelic-expression which may be equivalent to duplication of an active allele [12]. Our findings regarding the functional relationship of *KCNK9* methylation/expression may be consistent with this theory, as we observed increased *KCNK9*-expression together with relative hypomethylation at functional loci. We also observed a similar relationship for *KCNK5*. There is scant prior data on *KCNK12* expression in BC and Williams et al. suggested no alteration in their study [3]; we found *KCNK12* overexpression to be associated with TN subtype. Williams et al. described overexpression of *KCNK6*/*KCNK15*; we found that these were specifically overexpressed in luminal-A subtype.

Our findings are consistent with prior reports suggesting that some K2p-channels may be novel molecular-markers with differential expression related to biologically-aggressive TNBC, and may hold promise for innovative oncologic therapeutic/prevention strategies, as experience gained with pharmacological manipulation of K+ channels in other pathologies, might facilitate their use as molecular-targets in precision-medicine [7]. Wallace et al. suggest that K+ channel activity demonstrates promise as a pharmacologic target in BC and may represent a mechanism through which phytoestrogens act (e.g. Genistein) [18]. A recent study showed that inhibition of the intermediate-conductance calcium-activated K+ channel *KCNN3* with specific blockers including the antifungal clotrimazole, suppressed cell proliferation, migration and epithelial–mesenchymal transition in TNBC cells, and illustrates the potential of K+ channels as novel targets [17]. Sun et al. demonstrated that a monoclonal antibody (Y4) against *KCNK9* extracellular-domain effectively inhibits growth of human lung cancer xenografts and BC metastasis in mice, and suggest that antibody-based *KCNK9* targeting is a promising therapeutic strategy in *KCNK9* overexpressing malignancies [32]. Skaar et al. have been researching whether loss of *KCNK9* imprint-control and/or differential methylation could be adapted in novel approaches for clinical diagnosis and targeted therapy for TNBC [11–14].

## Conclusions

In summary, our results suggest that several K2p-channel methylation/expression markers are related to TNBC. *KCNK5* and *KCNK9* show overexpression and relative hypomethylation of specific CpG loci, and have negative methylation/gene-expression correlations, which are features that support functional biology. Study strengths include: TCGA is a large, well characterized BC resource; we used the target tumor-tissue of interest, not peripheral blood; and our findings are consistent with, and build on, other reports from TCGA. Our findings are considered preliminary but may lead to further exploration of K2p-channels as potential molecular-targets in precision-medicine, as pharmacological manipulation of K+ channels is currently feasible.

## Limitations

TCGA is not a population-based study or clinical trial and may be subject to selection bias. There was missing data on CpG loci with null-reads which may have been excluded due to quality-control protocols. Missing data may cause loss of analytic precision and power and could potentially bias results [33]. Our analysis used specific CpG probes present on the 450K array, but a more comprehensive fine-mapping sequencing approach could facilitate interrogation of additional loci. Potential modeling inadequacies may also result from failure to include molecular-markers not available to us, such as protein-expression, non-coding RNAs and expression-quantitative-trait-loci. K2p copy-number-change may also be important and this analysis is planned as a future study. Regarding survival analysis, specific treatment data is unavailable, and since follow-up time and number of survival events are limited, we were unable to examine prognosis, as our study is largely under-powered to detect differences between groups. Regarding sensitivity analyses, overlap between the RSEM and Agilent expression-arrays was only 499 cases, and as such sampling differences could account for observed variation in expression profiling. These limitations make the present study results preliminary in nature and our findings require validation in additional studies.

## Additional files

**Additional file 1: Table S1.** Distribution of K2p gene expression for selected factors.

**Additional file 2: Table S2.** Distribution of K2p gene expression status for subtype outcomes by assay type.

**Additional file 3: Table S3.** Association of CpG loci with triple negative subtype.

**Additional file 4: Table S4.** Annotation and expression correlation for all K2p methylation loci associated with triple negative subtype.

## Abbreviations

K2p: 2-pore domain potassium
K+: potassium
BC: breast cancer
TN: triple-negative
TCGA: The Cancer Genome Atlas
ERE: estrogen response-elements
nH: non-hispanic
ER/PR: estrogen/progesterone receptor
HER2: human epidermal growth factor receptor-2
GpG: cytosine–guanine
UIC: University of Illinois at Chicago
IRB: Institutional Review Board

## References

[1] Patel AJ, Lazdunski M (2004). The 2P-domain K+ channels: role in apoptosis and tumorigenesis. Pflugers Arch.

[2] Enyedi P, Czirjak G (2010). Molecular background of leak K+ currents: two-pore domain potassium channels. Physiol Rev.

[3] Williams S, Bateman A, O’Kelly I (2013). Altered expression of two-pore domain potassium (K2P) channels in cancer. PLoS ONE.

[4] Comes N, Serrano-Albarras A, Capera J, Serrano-Novillo C, Condom E, Ramon YCS, Ferreres JC, Felipe A (2015). Involvement of potassium channels in the progression of cancer to a more malignant phenotype. Biochem Biophys Acta.

[5] Kondratskyi A, Kondratska K, Skryma R, Prevarskaya N (2015). Ion channels in the regulation of apoptosis. Biochem Biophys Acta.

[6] Lastraioli E, Iorio J, Arcangeli A (2015). Ion channel expression as promising cancer biomarker. Biochem Biophys Acta.

[7] Pardo LA, Stuhmer W (2014). The roles of K(+) channels in cancer. Nat Rev Cancer.

[8] Alvarez-Baron CP, Jonsson P, Thomas C, Dryer SE, Williams C (2011). The two-pore domain potassium channel KCNK5: induction by estrogen receptor alpha and role in proliferation of breast cancer cells. Mol Endocrinol (Baltimore, Md).

[9] Clarke C, Madden SF, Doolan P, Aherne ST, Joyce H, O’Driscoll L, Gallagher WM, Hennessy BT, Moriarty M, Crown J (2013). Correlating transcriptional networks to breast cancer survival: a large-scale coexpression analysis. Carcinogenesis.

[10] Conway K, Edmiston SN, Tse CK, Bryant C, Kuan PF, Hair BY, Parrish EA, May R, Swift-Scanlan T (2015). Racial variation in breast tumor promoter methylation in the carolina breast cancer study. Cancer Epidemiol Biomark Prev.

[11] Luedi PP, Dietrich FS, Weidman JR, Bosko JM, Jirtle RL, Hartemink AJ (2007). Computational and experimental identification of novel human imprinted genes. Genome Res.

[12] Skaar D, Gould M, Jirtle R, Seewaldt V (2014). Altered imprinted gene DMR regulatory methylation in breast cancer. Cancer Epidemiol Biomark Prev.

[13] Desai S, Skaar D, Dietze E, Ambrose A, Jirtle R, Seewaldt V (2014). Abstract C65: loss of imprinting of KCNK9 in African American women with triple-negative breast cancer. Cancer Epidemiol Biomark Prev.

[14] Skaar DA, Seewaldt VL, Gould MN, Jirtle RL (2012). Abstract 4043: loss of imprinting of KCNK9 in breast cancer. Can Res.

[15] Mu D, Chen L, Zhang X, See LH, Koch CM, Yen C, Tong JJ, Spiegel L, Nguyen KC, Servoss A (2003). Genomic amplification and oncogenic properties of the KCNK9 potassium channel gene. Cancer Cell.

[16] Riener MO, Nikolopoulos E, Herr A, Wild PJ, Hausmann M, Wiech T, Orlowska-Volk M, Lassmann S, Walch A, Werner M (2008). Microarray comparative genomic hybridization analysis of tubular breast carcinoma shows recurrent loss of the CDH13 locus on 16q. Hum Pathol.

[17] Zhang P, Yang X, Yin Q, Yi J, Shen W, Zhao L, Zhu Z, Liu J (2016). Inhibition of SK4 potassium channels suppresses cell proliferation, migration and the epithelial–mesenchymal transition in triple-negative breast cancer cells. PLoS ONE.

[18] Wallace JL, Gow IF, Warnock M (2011). The life and death of breast cancer cells: proposing a role for the effects of phytoestrogens on potassium channels. J Membr Biol.

[19] Cerami E, Gao J, Dogrusoz U, Gross BE, Sumer SO, Aksoy BA, Jacobsen A, Byrne CJ, Heuer ML, Larsson E (2012). The cBio cancer genomics portal: an open platform for exploring multidimensional cancer genomics data. Cancer Discov.

[20] Gao J, Aksoy BA, Dogrusoz U, Dresdner G, Gross B, Sumer SO, Sun Y, Jacobsen A, Sinha R, Larsson E (2013). Integrative analysis of complex cancer genomics and clinical profiles using the cBioPortal. Sci Signal.

[21] Cancer-Genome-Atlas-Network (2012). Comprehensive molecular portraits of human breast tumours. Nature.

[22] Tomczak K, Czerwinska P, Wiznerowicz M (2015). The cancer genome atlas (TCGA): an immeasurable source of knowledge. Contemp oncol (Poznan, Poland).

[23] Illumina. Infinium HumanMethylation450K BeadChip. 2017; Array Support. https://support.illumina.com/array/array_kits/infinium_humanmethylation450_beadchip_kit.html. Accessed 09 Aug 2017.

[24] Li B, Dewey CN (2011). RSEM: accurate transcript quantification from RNA-Seq data with or without a reference genome. BMC Bioinform.

[25] Kent WJ, Sugnet CW, Furey TS, Roskin KM, Pringle TH, Zahler AM, Haussler D (2002). The human genome browser at UCSC. Genome Res.

[26] Ali H, Rueda OM, Chin SF, Curtis C, Dunning MJ, Aparicio S, Caldas C (2014). Genome-driven integrated classification of breast cancer validated in over 7500 samples. Genome Biol.

[27] Curtis C, Shah SP, Chin SF, Turashvili G, Rueda OM, Dunning MJ, Speed D, Lynch AG, Samarajiwa S, Yuan Y (2012). The genomic and transcriptomic architecture of 2000 breast tumours reveals novel subgroups. Nature.

[28] Dawson SJ, Rueda OM, Aparicio S, Caldas C (2013). A new genome-driven integrated classification of breast cancer and its implications. EMBO J.

[29] Tell RW, Horvath CM (2014). Bioinformatic analysis reveals a pattern of STAT3-associated gene expression specific to basal-like breast cancers in human tumors. Proc Natl Acad Sci.

[30] Koch A, De Meyer T, Jeschke J, Van Criekinge W (2015). MEXPRESS: visualizing expression, DNA methylation and clinical TCGA data. BMC Genom.

[31] Pei L, Wiser O, Slavin A, Mu D, Powers S, Jan LY, Hoey T (2003). Oncogenic potential of TASK3 (Kcnk9) depends on K+ channel function. Proc Natl Acad Sci USA.

[32] Sun H, Luo L, Lal B, Ma X, Chen L, Hann CL, Fulton AM, Leahy DJ, Laterra J, Li M (2016). A monoclonal antibody against KCNK9K(+) channel extracellular domain inhibits tumour growth and metastasis. Nat commun.

[33] Sterne JA, White IR, Carlin JB, Spratt M, Royston P, Kenward MG, Wood AM, Carpenter JR (2009). Multiple imputation for missing data in epidemiological and clinical research: potential and pitfalls. BMJ.

